# Protective Effect of Cinnamaldehyde on METH-induced Neurotoxicity in PC12 Cells via Inhibition of Apoptotic Response and Oxidative Stress

**DOI:** 10.22037/ijpr.2020.111891.13411

**Published:** 2021

**Authors:** Roghayeh Rashidi, Seyed Adel Moallem, Mohammad Moshiri, Farzin Hadizadeh, Leila Etemad

**Affiliations:** a *Pharmacological Research Center of Medicinal Plants, Mashhad University of Medical Sciences, Mashhad, Iran.*; b *Department of Pharmacodynamics and Toxicology, School of Pharmacy, Mashhad University of Medical Sciences, Mashhad, Iran. *; c *Department of Pharmacology and Toxicology, Faculty of Pharmacy, University of Ahl Al Bayt, Karbala, Iraq. *; d *Medical Toxicology Research Center, Mashhad University of Medical Sciences, Mashhad, Iran* *.*; e *Department of Clinical Toxicology, Imam Reza Hospital, Mashhad University of Medical Sciences, Mashhad, Iran* *.*; f *Biotechnology Research Center, Pharmaceutical Technology Institute, Mashhad University of Medical Sciences, Mashhad, Iran. *; g *Department of Medicinal Chemistry, School of Pharmacy, Mashhad University of Medical Sciences, Mashhad, Iran.*; h *Pharmaceutical Research Center, Pharmaceutical Technology Institute, Mashhad University of Medical Sciences, Mashhad, Iran.*

**Keywords:** Amphetamine, Cinnamaldehyde, Cinnamon, Methamphetamine, Neurotoxicity

## Abstract

Methamphetamine (METH) is a potent central nervous system (CNS) stimulant and frequently used illegal drugs. Repeated exposure to METH can induce degenerative changes in dopaminergic and serotonergic axons. There is no standard medical treatment for METH’s neurotoxic effects. Cinnamaldehyde is an important compound of cinnamon and has activities against neurological disorders. The present study was designed to examine the neuroprotective effect of *trans*-cinnamaldehyde (TCA) on METH-induced cytotoxicity. PC12 cells were treated with METH (2.5 mM) 24 h after treated with different concentrations of TCA (3.75- 50 μM). The percentage of cell survival was evaluated by MTT assay and the following parameters were measured to detect apoptosis and oxidative stress responses: DNA fragmentation, ROS production and GSH content. Exposure to 2.5 mM METH decreased the cell viability and GSH levels, caused the generation of reactive oxygen species and ultimately induced apoptosis. Pretreatment with TCA at 3.125-25 μM significantly attenuated cell viability loss. TCA, especially at a concentration of 12.5 and 25 μM, decreased the apoptosis and ROS generation and increased the GSH level compared with the METH group. The findings of the present study suggested that TCA exerted a protective effect against METH-induced neurotoxicity through mechanisms related to antioxidant and anti-apoptosis. It is suggested that TCA may be useful for the prevention and treatment of harmful effects of METH on the brain.

## Introduction

*Methamphetamine* (*N-*methyl-1-phenylpropan-2-amine or METH) is a potent central nervous system (CNS) stimulant and frequently used illegal. In tablet form (Desoxyn^®^), it was used to treat psychophysiological disorders such as Attention-Deficit/Hyperactivity Disorder (ADHD) and obesity and narcolepsy in the 1940s (1). METH is a lipophilic cationic molecule that can cross the blood-brain barrier and exert neurotoxic effects on the CNS (2, 3). Studies in human addicts have revealed that repeated exposure to amphetamines can induce degenerative changes in dopaminergic and serotonergic axons (4). Different hypotheses about the mechanism responsible for METH-induced neurotoxicity have been suggested (5, 6). METH penetrates the neuron and displays both dopamine and serotonin in the vesicles, which led to the release of a large amount of them into the synaptic cleft (2). It can enter the intracellular organelles such as mitochondria and vesicles and alters the electrochemical gradient and enzyme activity (7). Dopamine auto-oxidation, synthesis and metabolism generate reactive oxygen species such as hydrogen peroxide, hydroxyl superoxide and nitrogen radicals (8). It has been further shown that the generation of reactive radicals is the leading cause of mitochondrial dysfunction, decreased energy metabolism and apoptosis (9). Inhibition of electron transport chain, excitotoxicity, oxidative stress, inflammation and apoptosis all play a critical role in METH induced neurotoxicity (10). There is no standard medical treatment for METH’s neurotoxic effects (1).

Cinnamaldehyde (CA) is the most important organic compound that is present in the essential oil from the stem bark of *Cinnamomum cassia* and gives its flavor and odor (11). The therapeutic effects of cinnamon are mainly attributed to its cinnamaldehyde derivatives, cinnamyl and eugenol. Various biological activities are related to CA include: antidiabetic, antifungal, anticancer, anti-inflammatory and antimicrobial (12). In addition, it has been reported that cinnamaldehyde has activities against neurological disorders, such as Parkinson’s and Alzheimer’s diseases (13-15). In addition, some reports have shown that *trans*-cinnamaldehyde (TCA) has potent anti-inflammatory activity in the central nervous system (16). The present study was designed to examine the neuroprotective effect of TCA on METH-induced cytotoxicity.

## Experimental

*Materials*

RPMI 1640 medium and fetal bovine serum (FBS) were purchased from Invitrogen. 3 (4,5-dimethylthiazol-2-yl)-2,5-diphenyl tetrazolium (MTT), propidium iodide (PI) and fluorescent probe 2,7-dichlorofluorescein diacetate (DCF-DA) were provided from Sigma Chemical Co.TCA was prepared from Sinopharm Chemical Reagent Co, Ltd. 

*Cell Culture*

PC12 cells were cultured in RPMI medium (Bioidea, Iran) containing 100 U/mL penicillin and streptomycin, supplemented with 10% fetal bovine serum (FBS). The cells were maintained in a humid incubator at 37 °C and 5% CO_2._

*Cell Viability*

3-(4,5-dimethylthiazol- 2-yl)-2,5-diphenyltetrazolium bromide (MTT) colorimetric assay was used to measure cellular viability and activation (17). In brief, PC12 cells were cultured in a 96-well microplate at a density of 5000 cells/well. To determine an approperiate concentrations, the cells were incubated with a fresh medium containing 0, 0.5,1, 1.5, 2, 2.5 and 3 mM of METH or 0, 25, 50, 75 100, 150 and 200 µM of TCA for 24 h. At the next step, cells were exposed to selected concentrations of TCA (3.125, 6.25, 15, 30 and 50 µM) for 24 h. Then METH at a final concentration of 2.5 mM was added to each well. After incubation for 24 hours, MTT solution at a final concentration of 0.5 mg/mL was added to the well. Then, the media was removed, and the formazan crystals were dissolved in dimethylsulfoxide (DMSO). The absorbance of the purple solution was measured at 545 nm (630 nm as a reference) in an ELISA reader (Start Fax-2100, UK).

*PI staining*

The apoptotic effect was detected by PI staining, which determines apoptotic cells in the sub-G1 peak on the DNA content histogram (18, 19). Briefly, PC12 cells were seeded at 5 × 10^4 ^cells per well in a 12-well plate. Then TCA (12.5 and 25 µM) and METH (2.5 mM) were added to each well at two (2) consecutive 24 h periods, respectively. After then, suspensions of single cells were prepared by trypsinization and washed twice with cold PBS. The pellet cells were resuspended in PBS containing 50 μg/mL PI and 150 U/mL RNAse for 45 minutes at 37 ºC. The treated cells were then subjected to FACS flow cytometry analysis (20).

*Measurement of intracellular ROS*

The 2’,7’-dichlorofluorescein diacetate (DCFH2-DA) is a cell-permeable fluorimetric probe for detecting reactive oxygen (19). Upon reaction of DCFH with ROS, the highly fluorescent dichlorofluorescein (DCF) is produced. As explained before, PC12 cells were treated with different concentrations of TCA and METH. On the last day of the experiment, the medium was replaced with RPMI containing 10 µM of DCFH-DA and the cells were incubated for 30 min in the dark at room temperature. The intensity of fluorescence from the DCF was measured by Partec TM cytometry (Germany) (excitation wavelength 480 nm; emission wavelength 530 nm) (21).

*Reduced glutathione (GSH) assay*

GSH was measured via the formation of yellow color in the presence of DTNB [5,5’-di thiobis-(2-nitrobenzoic acid)]. Briefly, the day after the final treatment, cells were washed twice with phosphate-buffered saline (PBS) and then treated with 5% trichloroacetic acid incubated at 37 ºC for 30 min (22). After centrifugation at 14,000 g for 10 min, the supernatant was mixed again with 1 mL of a reaction mixture containing 0.1 M sodium phosphate buffer (pH 7.5), 0.6 mM DTNB at room temperature for 5 min. The absorbance of the solution was measured at 412 nm using a spectrophotometer (23). 

*Statistical Analysis*

Results are expressed as mean ± SD. One-way ANOVA followed by Tukey-Kramer was performed to compare the results. Differences were considered statistically significant when *P* < 0.05.

## Results

*Effects on cell viability*

**Figure 1:**
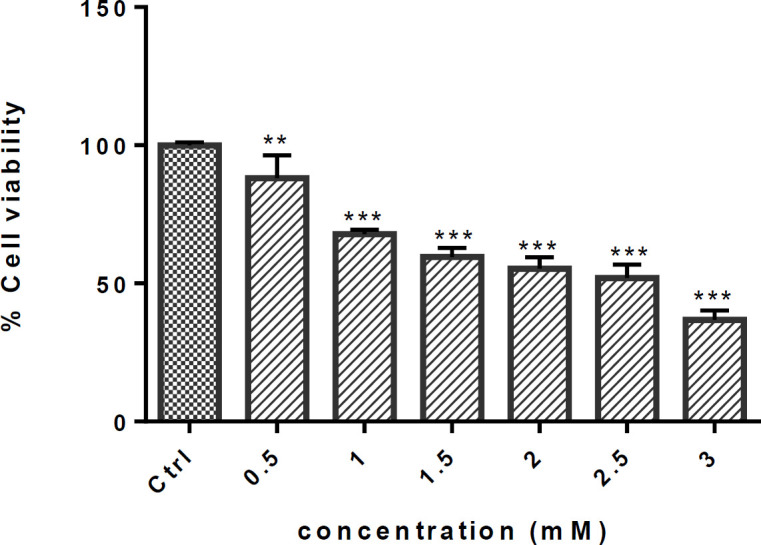
Effect of METH on cell viability of PC12 cells. Cells were treated with different concentrations of METH for 24h. Viability was quantitated by MTT assay. Data are expressed as mean ± SEM of six separate experiments. ^**^P <0.01 and ^***^P < 0.001 *vs.* control group

Exposure to METH significantly decreased the cell viability at all concentrations in a dose-dependent manner, as shown in Figure 1. Cell viability (expressed as the percentage to the control) reduced by 50% when exposed to 2.5 mM METH (*P* < 0.001) (Figure 1). 

**Figure 2:**
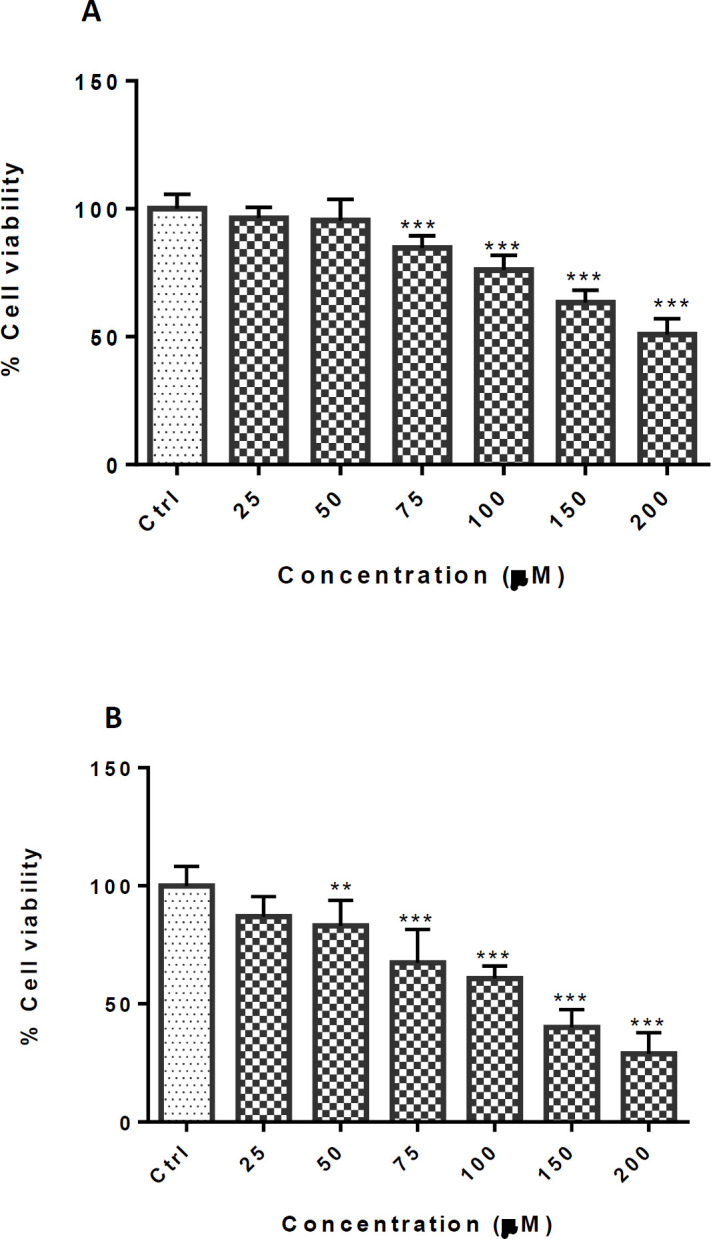
Effect of TCA on cell viability of PC12 cells. Cells were treated with different TCA concentrations for (A) 24 and (B) 48 h. Viability was quantitated by MTT assay. Data are expressed as mean ± SEM of six separate experiments. ^**^P <0.01 and ^***^P < 0.001 *vs.* control group

In order to assess the nontoxic cinnamaldehyde concentration, the cells were treated with different concentrations (0-200 µM) for 24 and 48 h. There were no significant differences between the group that received TCA at 25 and 50 µM at 24 h exposure and 25 µM at 48 h exposure, compared with the control group. The IC_50_ (50% inhibitory concentration) was calculated at 200 µM and 125 µM after 24 and 48 h exposure, respectively (Figure 2). According to the results, the protective effect of TCA at a concentration below 50 µM was evaluated against METH at a concentration of 2.5 mM. 

**Figure 3:**
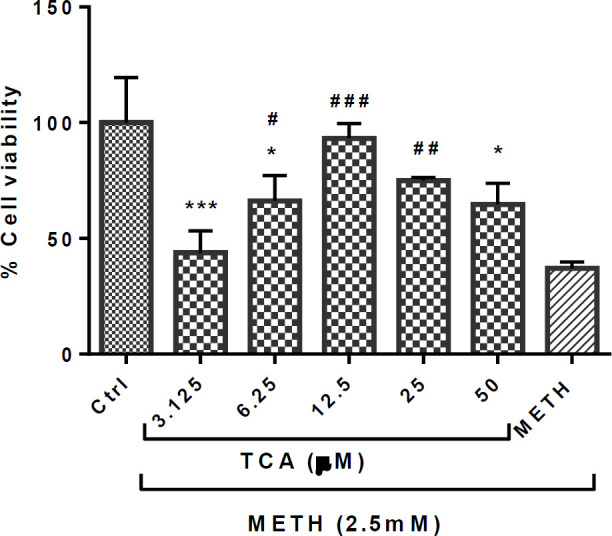
Effect of TCA on the METH-induced reductions in PC12 cell viability. Cell viability was assessed by MTT assay. PC12 cells were treated with TCA (3.125, 6.25, 12.5, 25 and 50 µM) for 24 h in the presence or absence of METH (2.5 mM). Data are expressed as mean ± SEM of six separate experiments. ^*^P < 0.05 and ^***^P < 0.001 *vs.* control group, ^#^*P *< 0.05, ^##^*P *< 0.01 and ^###^*P *< 0.001 *vs.* METH treated group

The results showed a significant increase in the cell viability of cells pretreated with TCA (6.25, 12.5 and 25 µM), compared with the METH group (*P* < 0.05, *P < *0.001 and *P* < 0.01, respectively), as shown in Figure 3. However, TCA only at 12.5 and 25 µM enhanced the PC12 proliferation to return to a normal level.

*Effect of TCA on METH-induced apoptosis*

**Figure 4.**
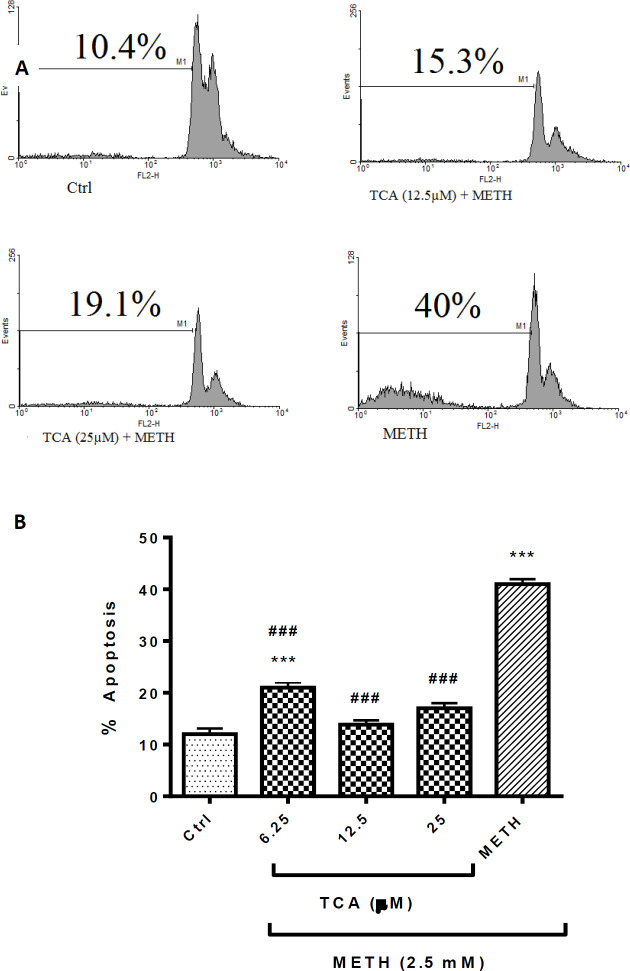
(A) Flow cytometry histograms of apoptosis assays by the PI method in PC12 cells Cells were treated with TCA (12.5 and 25 µM) for 24 h in the presence or absence of METH (2.5 mM). (B) The bar chart illustrates data as mean ± SEM of six separate experiments. ^***^P < 0.001 *vs. *control group, ^###^*P<* 0.001 *vs.* METH treated group

Quantitative analysis of apoptotic cells was measured with PI staining. Percentage of apoptotic cells increased to approximately 30% after 24 h incubation with METH (2.5 mM) over control (*P* < 0.001). Pretreatment of cells with 6.25, 12.5 or 25 µM of TCA significantly decreased the sub-G1 peak in flow cytometry histogram to 24.7% and 20.9%, respectively, in comparison with the METH group (*P* < 0.001) (Figure 4). There were no significant differences between TCA at 12.5 or 25 µM+METH and the control group.

*Effect of TCA on METH-induced ROS production*

**Figure 5:**
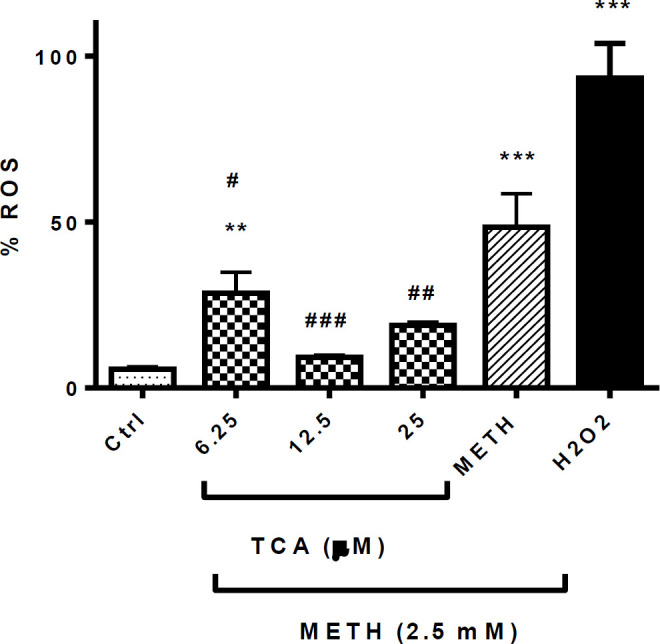
Effect of TCA on METH-induced ROS generation. Cell viability was assessed by MTT assay. PC12 cells were treated with TCA (12.5 and 25 µM) for 24 h in the presence or absence of METH (2.5 mM). Reactive oxygen species were measured using DCF-DA by flow cytometric analysis. Data are expressed as the mean ± SEM of six separate experiments. ^**^P < 0.01 and ^***^P < 0.001 *vs. *Control, ^#^*P* < 0.05, ^##^*P* < 0.01 and ^###^*P* < 0.001 *vs. *METH treated groups

After 24 h of exposure, METH and H2O2 significantly increased ROS generation in comparison to control (*P* < 0.001). Pretreatment with 6.25, 12.5 or 25 µM of TCA significantly decreased the level of intracellular ROS in comparison with the METH group (*P* < 0.001, *P *< 0.01 and *P* < 0.05, respectively) (Figure 5). However, exposure to the lowest concentration did not reduce the ROS to the normal level.

*Effect of TCA on METH-decreased GSH level*

**Figure 6:**
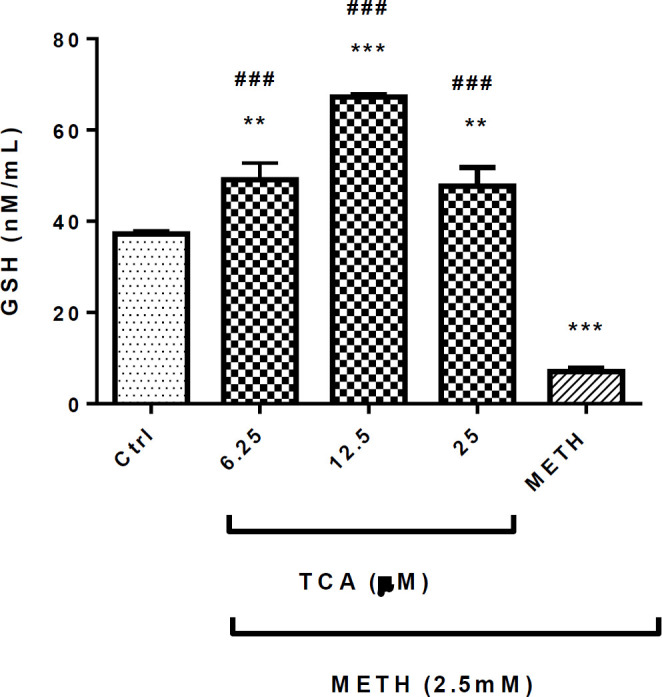
Effect of TCA on METH-decreased GSH generation. Cell viability was assessed by MTT assay. PC12 cells were treated with TCA (12.5 and 25 µM) for 24 h in the presence or absence of METH (2.5 mM). Data are expressed as the mean ± SEM of six separate experiments. ^***^P < 0.001 and ^**^P < 0.01*vs.* control, ^###^*P* < 0.001 *vs.* METH treated cells

METH (2.5 mM) significantly decreased the GSH level in comparison with the control group (*P* < 0.001). Pretreatment of cells with 6.25, 12.5 or 25 µM of TCA significantly increased the content of GSH (*P* < 0.001) in comparison with the METH group (Figure 6).

## Discussion

The current study demonstrated for the first time that TCA, extracted from cinnamon, exerts significant neuroprotective effects against METH-induced cytotoxicity by inhibiting apoptotic DNA fragmentation and ROS generation and increasing glutathione levels in PC12 cells. Continued METH abuse is widely believed to cause serotonergic and dopaminergic neuronal injury. The effects of abuse of METH include memory problems, aggression, psychotic and behavioral abnormalities (9, 24). Evidence suggests that multiple molecular and cellular mechanisms are involved in METH-induced CNS toxicity, such as oxidative stress, mitochondrial dysfunction, excitotoxicity and inflammatory responses (25). Previous studies indicated that exposure to METH at doses greater than 2mM might result in decreased PC12 cell viability (26, 27). Our data showed that METH-induced a dose-dependent reduction in cell viability, while TCA suppressed the cytotoxicity at the concentrations less-than-or-equal-to 25 µM. 

Both the *in-vivo* and *in-vitro* experimental findings indicated that cell apoptosis has a key role in METH neurotoxicity. They showed that it could be mediated by activation of the effector caspases 3, 9 and 11 and downregulation of Bcl-2/Bax expression (28-32). In this study, the quantitative analysis of apoptosis revealed a significantly higher rate of PC12 cell death after treatment with METH that was abolished by TCA pretreatment. Pretreatment with cinnamaldehyde (5, 10 and 20 μM) Could remarkably increase the cell viability and decrease the apoptosis by suppressing cytochrome c release, reducing the activity of caspase 3 and 9 in PC12 cells treated with glutamate. Exposure to CA also attenuated the glutamate-induced oxidative stress with decreased ROS and malondialdehyde (MDA) production and increased GSH level, which was in agreement with our study (33). The amount of GSH significantly decreased in TCA+METH treated group. In addition, the generation of ROS was inhibited by TCA treatment. The animal studies result supports the beneficial effects of cinnamaldehyde on brain damage and oxidative stress induced by a high-fat diet. The HFD increased MDA in serum and the brain and NO level in the cerebellum that was prevented by CA (34). The previous *in-vivo *and *in-vitro* studies confirmed the capacity of TCA and its metabolite, sodium benzoate, on reducing cell death, inflammation and oxidative stress (14, 35-38). However, several researchers have pointed out TCA can induce apoptosis in cancer cell lines at concentrations greater than 30µM (22, 39 and 40). In our study, TCA showed a protective effect against METH at a concentration of 25 µM. TCA significantly decreased the cell viability at higher concentrations and its effect was not in a dose-dependent manner. It may be due to differences in the used concentration of TCA and kind of cell lines.

## Conclusion

The findings of the present study suggested that TCA exerted a protective effect against METH-induced neurotoxicity through mechanisms related to anti-oxidation and anti-apoptosis. It is suggested that TCA may be useful for the prevention and treatment of harmful effects of METH on the brain.

## References

[1] Ghadiri A, Etemad L, Moshiri M, Moallem SA, Jafarian AH, Hadizadeh F, Seifi M (2017). Exploring the effect of intravenous lipid emulsion in acute methamphetamine toxicity. Iran. J. Basic Med. Sci..

[2] Sekine Y, Minabe Y, Ouchi Y, Takei N, Iyo M, Nakamura K, Suzuki K, Tsukada H, Okada H, Yoshikawa E, Futatsubashi M, Mori N (2003). Association of dopamine transporter loss in the orbitofrontal and dorsolateral prefrontal cortices with methamphetamine-related psychiatric symptoms. Am. J. Psychiatry.

[3] Davidson C, Gow AJ, Lee TH, Ellinwood EH (2001). Methamphetamine neurotoxicity: necrotic and apoptotic mechanisms and relevance to human abuse and treatment. Brain Res. Brain Res. Rev..

[4] Jiménez A, Jordà EG, Verdaguer E, Pubill D, Sureda FX, Canudas AM, Escubedo E, Camarasa J, Camins A, Pallàs M (2004). Neurotoxicity of amphetamine derivatives is mediated by caspase pathway activation in rat cerebellar granule cells. Toxicol. Appl. Pharmacol..

[5] Seiden LS, Sabol KE, Ricaurte GA (1993). Amphetamine: effects on catecholamine systems and behavior. Annu. Rev. Pharmacol. Toxicol..

[6] Seiden LS, Sabol KE (1996). Methamphetamine and methylenedioxymethamphetamine neurotoxicity: possible mechanisms of cell destruction. NIDA Res. Monogr..

[7] Gilgun-Sherki Y, Melamed E, Offen D (2001). Oxidative stress induced-neurodegenerative diseases: the need for antioxidants that penetrate the blood brain barrier. Neuropharmacology.

[8] Yamamoto B, Zhu W (1998). The effects of methamphetamineon the production of free radicals and oxidative stress. J. Pharmacol. Exp. Ther..

[9] Moshiri M, Hosseiniyan SM, Moallem SA, Hadizadeh F, Jafarian AH, Ghadiri A, Hoseini T, Seifi M, Etemad L (2018). The effects of vitamin B12 on the brain damages caused by methamphetamine in mice. Iran. J. Basic Med. Sci..

[10] Thrash B, Thiruchelvan K, Ahuja M, Suppiramaniam V, Dhanasekaran M (2009). Methamphetamine-induced neurotoxicity: the road to Parkinson’s disease. Pharmacol. Rep..

[11] Bakkali F, Averbeck S, Averbeck D, Idaomar M (2008). Biological effects of essential oils–a review. Food Chem. Toxicol..

[12] Wondrak GT, Villeneuve NF, Lamore SD, Bause AS, Jiang T, Zhang DD (2010). The cinnamon-derived dietary factor cinnamic aldehyde activates the Nrf2-dependent antioxidant response in human epithelial colon cells. Molecules.

[13] Pyo JH, Jeong YK, Yeo S, Lee JH, Jeong MY, Kim SH, Choi YG, Lim S (2013). Neuroprotective effect of trans-cinnamaldehyde on the 6-hydroxydopamine-induced dopaminergic injury. Biol. Pharm. Bull..

[14] Roghani M, Mehraein F, Zamani M, Negahdar F, Shojaee A (2017). The effects of aqueous cinnamon bark extract and cinnamaldehyde on neurons of substantia nigra and behavioral impairment in a mouse model of Parkinson’s disease. J. Basic Clin. Pathophysiol..

[15] George RC, Lew J, Graves DJ (2013). Interaction of cinnamaldehyde and epicatechin with tau: implications of beneficial effects in modulating Alzheimer’s disease pathogenesis. J. Alzheimers Dis..

[16] Chao LK, Hua KF, Hsu HY, Cheng SS, Lin IF, Chen CJ, Chen ST, Chang ST (2008). Cinnamaldehyde inhibits pro-inflammatory cytokines secretion from monocytes/macrophages through suppression of intracellular signaling. Food Chem. Toxicol..

[17] Mansouri M, Moallem S A, Asili J, Etemad L (2019). Cytotoxic and apoptotic effects of scrophularia umbrosa dumort extract on MCF-7 breast cancer and 3T3 cells. Rep. Biochem. Mol. Biol..

[18] de Lima TM, Amarante-Mendes GP, Curi R (2007). Docosahexaenoic acid enhances the toxic effect of imatinib on Bcr-Abl expressing HL-60 cells. Toxicol. In-vitro.

[19] Tayarani-Najaran Z, Mousavi S, Asili J, Emami S (2010). Growth-inhibitory effect of Scutellaria lindbergii in human cancer cell lines. Food Chem. Toxicol..

[20] Savitskiy VP, Shman TV, Potapnev MP (2003). Comparative measurement of spontaneous apoptosis in pediatric acute leukemia by different techniques. Cytometry B. Clin. Cytom..

[21] Rahimi VB, Askari VR, Emami SA, Tayarani-Najaran Z (2017). Anti-melanogenic activity of Viola odorata different extracts on B16F10 murine melanoma cells. Iran. J. Basic Med. Sci..

[22] Ka H, Park HJ, Jung HJ, Choi JW, Cho KS, Ha J, Lee KT (2003). Cinnamaldehyde induces apoptosis by ROS-mediated mitochondrial permeability transition in human promyelocytic leukemia HL-60 cells. Cancer Lett..

[23] Hosseinzadeh L, Malekshahi A, Ahmadi F, Emami SA, Hajialyani M, Mojarrab M (2018). The Protective Effect of Different Extracts of Three Artemisia Species against H2O2-Induced Oxidative Stress and Apoptosis in PC12 Neuronal Cells. Pharmacognosy Res..

[24] Riddle EL, Fleckenstein AE, Hanson GR (2006). Mechanisms of methamphetamine-induced dopaminergic neurotoxicity. AAPS J..

[25] Krasnova IN, Cadet JL (2009). Methamphetamine toxicity and messengers of death. Brain Res. Rev..

[26] Xiong Q, Ru Q, Tian X, Zhou M, Chen L, Li Y, Li C (2018). Krill oil protects PC12 cells against methamphetamine-induced neurotoxicity by inhibiting apoptotic response and oxidative stress. Nutr. Res..

[27] Tian X, Ru Q, Xiong Q, Yue K, Chen L, Ma B, Gan W, Si Y, Xiao H, Li C (2017). Neurotoxicity induced by methamphetamine-heroin combination in PC12 cells. Neurosci. Lett..

[28] Choi HJ, Yoo TM, Chung SY, Yang JS, Kim JI, Ha ES, Hwang O (2002). Methamphetamine-induced apoptosis in a CNS-derived catecholaminergic cell line. Mol Cells.

[29] Deng X, Cai NS, McCoy MT, Chen W, Trush MA, Cadet JL (2002). Methamphetamine induces apoptosis in an immortalized rat striatal cell line by activating the mitochondrial cell death pathway. Neuropharmacology.

[30] Genc K, Genc S, Kizildag S, Sonmez U, Yilmaz O, Tugyan K, Ergur B, Sonmez A, Buldan Z (2003). Methamphetamine induces oligodendroglial cell death in vitro. Brain Res..

[31] Jayanthi S, Deng X, Bordelon M, McCoy MT, Cadet JL (2001). Methamphetamine causes differential regulation of pro-death and anti-death Bcl-2 genes in the mouse neocortex. FASEB J..

[32] Wongprayoon P, Govitrapong P (2017). Melatonin protects SH-SY5Y neuronal cells against methamphetamine-induced endoplasmic reticulum stress and apoptotic cell death. Neurotox. Res..

[33] Lv C, Yuan X, Zeng HW, Liu RH, Zhang WD (2017). Protective effect of cinnamaldehyde against glutamate-induced oxidative stress and apoptosis in PC12 cells. Eur. J. Pharmacol..

[34] Ataiea Z, Mehrani H, Ghasemi A, Farrokhfall K (2019). Cinnamaldehyde has beneficial effects against oxidative stress and nitric oxide metabolites in the brain of aged rats fed with long-term, high-fat diet. J. Funct. Foods.

[35] Qi X, Zhou R, Liu Y, Wang J, Zhang WN, Tan HR, Niu Y, Sun T, Li YX, Yu JQ (2016). Trans-cinnamaldehyde protected PC12 cells against oxygen and glucose deprivation/reperfusion (OGD/R)-induced injury via anti-apoptosis and anti-oxidative stress. Mol. Cell Biochem..

[36] Fu Y, Yang P, Zhao Y, Zhang L, Zhang Z, Dong X, Wu Z, Xu Y, Chen Y (2017). trans-Cinnamaldehyde inhibits microglial activation and improves neuronal survival against neuroinflammation in BV2 microglial cells with lipopolysaccharide stimulation. Evid. Based Complement Alternat. Med..

[37] Pyo JH, Jeong YK, Yeo S, Lee JH, Jeong MY, Kim SH, Choi YG, Lim S (2013). Neuroprotective effect of trans-cinnamaldehyde on the 6-hydroxydopamine-induced dopaminergic injury. Biol. Pharm. Bull..

[38] Farag MR, Alagawany M, Tufarelli V (2017). In-vitro antioxidant activities of resveratrol, cinnamaldehyde and their synergistic effect against cyadox-induced cytotoxicity in rabbit erythrocytes. Drug Chem. Toxicol..

[39] Lin LT, Tai CJ, Chang SP, Chen JL, Wu SJ, Lin CC (2013). Cinnamaldehyde-induced apoptosis in human hepatoma PLC/PRF/5 cells involves the mitochondrial death pathway and is sensitive to inhibition by cyclosporin A and z-VAD-fmk. Anticancer Agents Med. Chem..

[40] Roth-Walter F, Moskovskich A, Gomez-Casado C, Diaz-Perales A, Oida K, Singer J, Kinaciyan T, Fuchs HC, Jensen-Jarolim E (2014). Immune suppressive effect of cinnamaldehyde due to inhibition of proliferation and induction of apoptosis in immune cells: implications in cancer. PLoS One.

